# Imaging When Acting: Picture but Not Word Cues Induce Action-Related Biases of Visual Attention

**DOI:** 10.3389/fpsyg.2012.00388

**Published:** 2012-10-15

**Authors:** Agnieszka Wykowska, Bernhard Hommel, Anna Schubö

**Affiliations:** ^1^Department of Psychology, Ludwig-Maximilians-Universität MünchenMunich, Germany; ^2^Department of Psychology, Leiden UniversityLeiden, Netherlands; ^3^Faculty of Psychology, Philipps-Universität MarburgMarburg, Germany

**Keywords:** action-perception links, intentional weighting, visual attention, ideomotor control, action representation

## Abstract

In line with the Theory of Event Coding (Hommel et al., 2001a), action planning has been shown to affect perceptual processing – an effect that has been attributed to a so-called *intentional weighting* mechanism (Wykowska et al., 2009; Memelink and Hommel, 2012), whose functional role is to provide information for open parameters of online action adjustment (Hommel, 2010). The aim of this study was to test whether different types of action representations induce intentional weighting to various degrees. To meet this aim, we introduced a paradigm in which participants performed a visual search task while preparing to grasp or to point. The to-be performed movement was signaled either by a picture of a required action or a word cue. We reasoned that picture cues might trigger a more concrete action representation that would be more likely to activate the intentional weighting of perceptual dimensions that provide information for online action control. In contrast, word cues were expected to trigger a more abstract action representation that would be less likely to induce intentional weighting. In two experiments, preparing for an action facilitated the processing of targets in an unrelated search task if they differed from distractors on a dimension that provided information for online action control. As predicted, however, this effect was observed only if action preparation was signaled by picture cues but not if it was signaled by word cues. We conclude that picture cues are more efficient than word cues in activating the intentional weighting of perceptual dimensions, presumably by specifying not only invariant characteristics of the planned action but also the dimensions of action-specific parameters.

## Introduction

Humans do not only react to exogenous stimuli but also – and in most of the cases – act voluntarily in accordance to their endogenous action planning. How do humans plan actions? How do human brains know how to act in order to achieve those plans? According to ideomotor views (e.g., Lotze, 1852; James, 1890; Greenwald, 1970; Prinz, 1987, 1997; Hommel et al., 2001a), action plans are represented in the form of anticipated sensory consequences of the planned actions. Through life-long experience with different types of actions, humans learn what sort of action consequences to expect. If the actual consequences of a given action do not meet the expected ones, this might elicit an error signal and the action might be modified accordingly (for a similar account, see forward models of e.g., Wolpert and Kawato, 1998; Wolpert and Ghahramani, 2000).

The idea that action planning relies on anticipations of sensory action effects implies a rather close link between action and perception. Indeed, there is increasing evidence for the common representation of perception- and action-related information (e.g., Prinz, 1997; Hommel et al., 2001a) and numerous findings have demonstrated bi-directional (sensorimotor and motor-sensory) interactions between action and perception (e.g., Müsseler, 1995; Deubel and Schneider, 1996; Müsseler and Hommel, 1997; Hommel, 1998, 2004; Craighero et al., 1999; Tucker and Ellis, 2001; Bekkering and Neggers, 2002; Schubö et al., 2004).

Evidence for bi-directional links between action and perception has been reported not only with the use of behavioral measures but also thanks to imaging techniques. For example, Schubotz and von Cramon (2002) showed – in an fMRI study – that specific areas of the premotor cortex were automatically activated during processing of stimuli’s perceptual attributes that were related to particular actions (when processing stimuli’s size, hand areas were activated; when processing pitch of tones, articulation areas showed enhanced activity). Similarly, Grèzes and Decety (2002) or Grafton et al. (1997) reported automatic activation of motor areas when objects with given affordances (Gibson, 1977) were only viewed. Finally, Handy et al. (2003), reported that an early sensory ERP component (P1) was modulated by (implicit) action relevance of stimuli, while Kiefer et al. (2011) showed modulations of the P1 related to stimuli that afforded the same action as an earlier presented prime.

If perception can be influenced by action planning no less than action is influenced by perception (Hommel, 2009, 2010), then one might consider action planning as yet another source of bias of perceptual processing. Hommel et al. (2001a) termed this mechanism *intentional weighting* (see also Wykowska et al., 2009; Hommel, 2010; Memelink and Hommel, 2012). According to the idea of intentional weighting, perceptual dimensions are weighted with respect to action relevance (intentionally weighted) in a similar manner as perceptual dimensions are weighted with respect to task demands (e.g., Müller et al., 2003; Wolfe et al., 2003) via a top-down dimensional set (e.g., Müller et al., 2009). According to Hommel (2010), the intentional weighting mechanism serves the purpose of channeling perceptual information through to open parameters of online action control processes.

Fagioli et al. (2007) provided evidence for a weighting mechanism that operates on perceptual dimensions in an oddball paradigm. In their study, participants were to detect size or location oddballs in a sequence of stimuli presented on a computer screen. At the same time, participants were asked to either point to or grasp an object located directly below the screen. The authors found that performance in location oddballs was better when participants intended to point as compared to grasp, whereas size oddball detection yielded better performance in the grasping condition. The authors concluded that perceptual dimensions were weighted with respect to action relevance, which resulted in the differential effects on performance.

Along similar lines, Wykowska et al. (2009) designed a paradigm in which participants were asked to perform a visual search task and a movement task. Importantly, these two tasks were completely unrelated both motorically and perceptually: the visual search display was presented on a computer screen and participants were searching for a size or luminance pop-out target among other items, arranged on a circular array. They responded with their dominant hand with one mouse key to target present displays and with the other key to target absent displays. The movement task was performed on items of a device positioned under the computer screen. The items of the device were also arranged on a circular array but were not spatially related to the items of the visual search task. Participants were asked to either grasp or point to one of the items with their other hand. A pictorial cue informed the observers about the type of movement they should prepare (but not execute until the search task is completed). Therefore, while the search display was presented and the search task was being performed, the action plan was supposed to-be held active. The authors assumed that grasp-size and point-luminance are two congruent action-perception pairs. In line with this assumption, they found that search reaction times depended on the prepared action: size detection was faster in the grasping condition relative to pointing, whereas luminance detection was faster in pointing relative to grasping. Wykowska et al. termed those effects action-perception congruency effects. Importantly, the study of Wykowska et al. (2009) showed that the intentional weighting mechanism can operate at already early stages of processing, such as pop-out detection.

These observations of an interaction between action planning and perception are consistent with previous demonstrations of perception-action interactions and support the idea that perceptual events and action plans are represented in a common representational domain. At the same time, however, they go beyond previous demonstrations by showing that preparing for an action can facilitate processing on an entire perceptual dimension (rather than the processing of particular feature values). Previous work has convincingly shown that feature overlap between perceived events and planned actions is beneficial. For instance, behavioral and ERP correlates of perceptual processes are modulated by the feature-related congruency between an *observed* and a prepared/executed action (e.g., Press et al., 2010; Bortoletto et al., 2011) and by congruency between actions and objects (e.g., Rizzolatti et al., 1994; Deubel and Schneider, 1996; Humphreys et al., 2010). This has been attributed to direct interactions between the feature codes representing stimulus events and action plans (Kornblum et al., 1990; Hommel et al., 2001a), which are also assumed to underlie stimulus-response compatibility effects in general. However, feature-code interactions cannot explain the observation that preparing for a particular type of action facilitates the processing of entire feature dimensions, as shown by Fagioli et al. (2007) and Wykowska et al. (2009). Explaining such effects requires the assumption that action planning affects the output gain associated with particular dimension maps, which determines the impact of any feature value falling onto the particular dimension (Wykowska et al., 2009; Hommel, 2010) – as suggested by the intentional weighting notion (Hommel et al., 2001a).

### Aim of study

**Figure 1:**
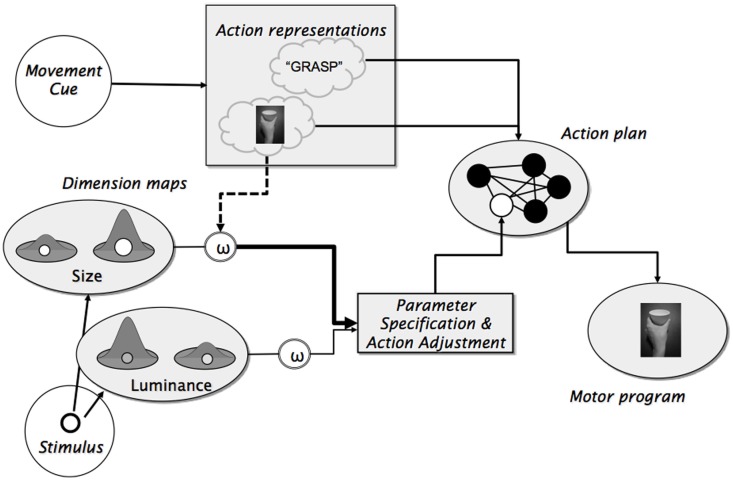
**Schematic representation of the idea that action planning not only determines the invariant aspects of an action but also biases attention toward feature dimensions that are likely to provide relevant information for open parameters of a given action (unfilled circle in the “Action plan” module)**.

The available evidence provides strong support for the idea that action planning can shape and systematically bias attentional selection, but the underlying mechanism is not yet well understood. According to Hommel (2010) and Wykowska et al. (2009), the attentional biases reflect top-down control from action planning processes. As shown in Figure 1, the idea is that action plans consist of pre-specified feedforward codes that determine the invariant (and commonly explicitly intended) aspects of a given action, such as the effector being used, the shape that the hand will need to assume, etc. (see Figure 1, the filled circles in the “Action plan”); and of the dimensions of the open parameters that are to-be filled in (later) online, such as information about the precise spatial location and possible obstacles (see Figure 1, the unfilled circle in the “Action plan”). The action plan takes effective control of the executed motor program. As Wykowska et al. (2009) and Hommel (2010) suggest, action representation might not only specify invariant action parameters of the action plan but also bias attention toward feature dimensions that are likely to provide sensory information that is suited to fill the open parameters. In Figure 1, this is indicated by the stippled line from the mental representation of the grasping action to the output of the size dimension map, which is more strongly weighted so to increase the impact of information from that map on parameter specification.

The idea that motivated the present study was that different kinds of action representations might trigger the dimensional weighting of action-relevant perceptual information to different degrees. Actions can be represented in different ways. According to ideomotor theorizing, an agent might (re-) create a visual image of a wanted action effect, such as that of her hand holding a particular cup. The success of the action would then be assessed by comparing this image with the actual visual outcome of the action, so that the action would only be judged successful if the image and visual outcome match to a sufficient degree. In our previous research, we have used visual action representations (i.e., pictures of a hand holding a cup) to cue the to-be-prepared action. Given that the preparation these cues have evoked was successful in biasing visual search in an unrelated task, it makes sense to assume that picture cues activate the intentional weighting mechanism, which increased the output gain for action-relevant feature maps. But what about verbal cues? However specific verbal description of the task might be, the resulting cognitive representation of the task is unlikely to be as closely related to particular perceptual dimensions. This need not impair the performance of the action, at least not the speed of initiating it (a process that is likely to rely on feedforward specifications rather than online control), but it might affect the degree to which perceptual feature dimensions are prepared for online control. In other words, word cues might be less potent to trigger the intentional weighting mechanism.

Converging evidence suggesting that verbal cues might be less effective in triggering the intentional weighting mechanism than picture cues comes from research on the impact of visual cues on attention (e.g., Wolfe et al., 2004; Vickery et al., 2005; Gibson and Kingstone, 2006). For example, Gibson and Kingstone (2006) observed that, in a spatial-cuing paradigm (Posner, 1980), 100%-valid central directional word cues were considerably less effective than other central cues, such as arrows, gaze, or peripheral cues. Referring to the distinction between projective and deictic spatial relations, the authors conclude that interpreting word cues might be more complex and less direct than other directional cues and therefore, might not trigger reflexive attentional orienting to the same extent as other (less indirect) cues. In a different paradigm, Vickery et al. (2005) examined the efficiency of word cues and picture cues in setting up a target template for a visual search task. The results showed that semantic cues were far less effective than other pictorial cues, even if the latter did not convey exact information about the target item. Evidence for the lesser potency of verbal stimuli has also been reported from research on stimulus-response compatibility (e.g., Proctor et al., 2009).

**Figure 2:**
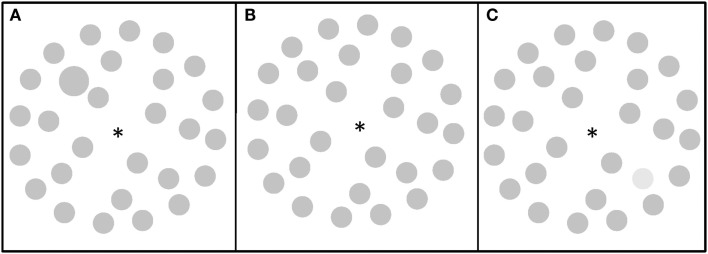
**Visual search displays**. **(A)** A visual search display containing a size target (Experiment 1 and 2); **(B)** a visual search display with no target (Experiment 1 and 2); **(C)** a visual search display containing a luminance target (Experiment 2).

If we assume that verbal action cues might be sufficient to properly plan the invariant feedforward components of an action but perhaps less efficient to prepare the system for the later intake of online information, it is possible that cross-task congruency effects as found by Fagioli et al. (2007) and Wykowska et al. (2009) are not observed or at least less pronounced if action planning is cued by verbal action descriptions rather than pictures. To test this possibility, the present study included not only pictorial cues but also word cues to inform participants about what type of movement to prepare. We employed a paradigm that was very similar to that used by Wykowska et al. (2009) and Wykowska et al. (2011). Participants were asked to perform two tasks: a visual search task for size targets and a movement task (grasping or pointing toward a paper cup). The main difference between the present and the previously used paradigms was that participants were informed regarding what action they should prepare by either picture or word cues (see Figure 2). As in Wykowska et al. (2009), we examined the congruency effect and thus tested whether faster reaction times would be obtained for targets in the visual search task that were congruent with a given action (size targets-grasping and luminance targets-pointing), relative to incongruent pairs (size targets-pointing and luminance targets-grasping); and whether these effects would be dependent on the type of movement cue.

## Experiment 1

### Method

#### Participants

Eighteen paid volunteers (11 women) aged from 20 to 35 years (mean age: 24) took part. All but three were right-handed; all of them reported normal or corrected-to-normal vision. The experiment was conducted with the understanding and consent of each participant.

#### Stimuli and apparatus

**Figure 3:**
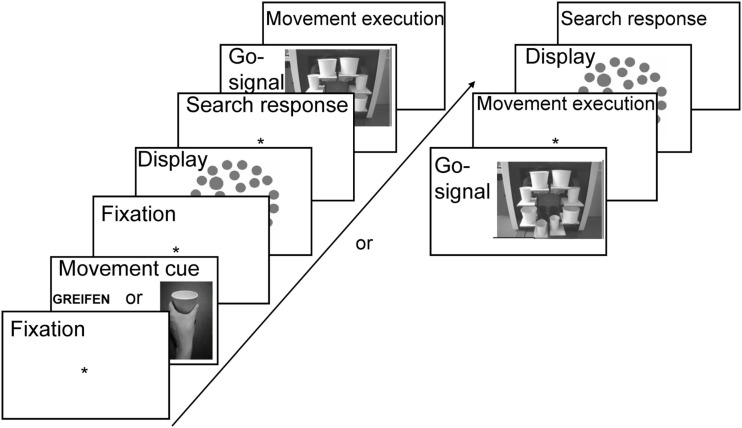
**Trial sequence in Experiment 1**. Either word or picture cues informed participants about the required action type. The order of search task and movement task were intermixed randomly. Participants either performed the search task and then the movement task (left) or first the movement task and then the search task (right).

Stimuli were presented on a 17″ CRT screen (100 Hz refresh rate) placed at a distance of 110 cm from a participant. Stimulus presentation was controlled by E-Prime presentation software (Psychology Software Tools, Pittsburgh, PA, USA). Cues specifying what type of action to prepare (i.e., grasping or pointing) consisted of either centrally presented German words “GREIFEN” (Grasp) or “ZEIGEN” (Point) covering 6.8° × 0.8° (“ZEIGEN”) or 7.9° × 0.8° (“GREIFEN”) of visual angle (each letter was of 0.8° × 0.8° size), or were photographs of a left hand performing a pointing or a grasping movement on a white paper cup, see Figure 3. The photographs were black and white covering 8.5° × 11.3° of visual angle.

The search display always contained 28 items (gray circles, 1.1° in diameter; 22 cd/m^2^ of luminance) positioned on three imaginary circles with a diameter of 3.4°, 7.4°, and 11.3°, see Figure 2. To simplify the design (due to a large number of other factors), the target was defined by only one dimension, i.e., size: a larger circle, 1.4° in diameter and could appear on one of six lateralized positions (three left, three right) on the middle circle. Participants were to detect the larger circle as the target in target present trials (50% of trials; one mouse key) and reject blank trials (50% of trials; the other mouse key).

The movement execution device (MED, see Figure 3) was placed at a distance of 80 cm from the participants’ seat. The midpoint of the device was situated 50 cm below and 30 cm in front of the computer screen. The MED consisted of a 43 cm × 54 cm × 13 cm box containing eight LEDs positioned on an imaginary circle of 22.2° in diameter. Slightly beneath each of the LEDs, rectangular cardboard pads were attached. White paper cups were positioned on those pads (see Figure 3) and covered the LEDs. All the cups had the same height (4.5°), weight (2 g), and luminance (3 cd/m^2^). They could only vary in diameter with four cups being larger (5.7°) and four smaller (4°). The LEDs behind the cups were lighting up the cups (luminance values of lit-up cups were equal to: 32 cd/m^2^).

#### Procedure

All participants took part in three sessions, one practice session and two subsequent experimental sessions with at least 1 day in between each of the sessions. In the practice session, participants performed two blocks of one movement type only (pointing or grasping, 48 trials per block) and one block of both types of movement randomly intermixed (64 trials). In this last block, participants were trained to perform 64 trials identical to those they would perform in the first experimental sessions proper. The two experimental sessions differed with respect to the type of movement cue. That is, in one session, the movement was signaled by a picture cue, in the other session by a word cue. Each of the experimental sessions consisted of 576 trials.

At the beginning of the experimental session, participants performed a short warm-up block (16 trials) in which they practiced the movements only. The movement task was randomized and participants were presented with a movement cue (word or picture) informing about the movement type they were to execute (cf. Figure 3). To ensure that the grasping/pointing action would be activated immediately after cue presentation (and not that participants would memorize the cue, and retrieve it only after completion of the search task), we randomized order of tasks (visual search first vs. movement first). Therefore, in 66% of trials, the search task was to-be performed first and only then the movement executed. In these trials, subsequent to the cue presentation and a blank display (300 ms), the search display was presented for 100 ms. Participants were asked to respond to the visual search task immediately by pressing the right/left mouse key with the index and middle finger of their right hand. Both speed and accuracy were stressed. In these trials, a go-signal occurred after the response to the visual search task, i.e., one of the LEDs on MED lit-up for 300 ms, which signaled that observers should execute the prepared movement with their left hand, and which cup they should point to or grasp from the side. Only accuracy was stressed in the movement task.

In the remaining 33% of trials, movement task was to-be executed immediately after the cue presentation (also signaled by the LED lighting up behind one of the cups), and only then, the visual search task was to-be performed. That is, subsequent to movement cue presentation and a blank display (500 ms), the go-signal occurred, and participants were asked to perform the movement task with their left hand. Only upon completion thereof, a visual search display was presented and participants were to perform the search task with their right hand. Also in these trials, speed and accuracy was stressed in the search task and only accuracy was stressed in the movement task. Correctness of movement execution was registered by the experimenter, who monitored participants with the use of a camera. For visualization of the entire trial sequence, see Figure 3.

#### Data analysis

Incorrect movement trials as well as outliers in the search task (±3 SD from mean RT for each participant and each block) were excluded from further analyses. From the remaining data, RTs in the detection task were submitted to analyses of variance (ANOVAs) with: *task order* (search first vs. movement first), *cue type* (word vs. picture), *movement type* (point vs. grasp), and *trial type* (target absent vs. target present trials) as within-subject factors. The order of cue type (word cues first vs. picture cues first) was a between-subject factor. The analysis of error rates in the movement task was performed with *task order* (search first vs. movement first)^1^, *cue type* (word vs. picture), *movement type* (point vs. grasp) as within-subjects factors. For the analyses of the error rates in the search task, incorrect trials in the movement task were excluded and individual mean error rates were submitted to analogous ANOVAs as in data analysis of RT data in the search task.

### Results

**Figure 4:**
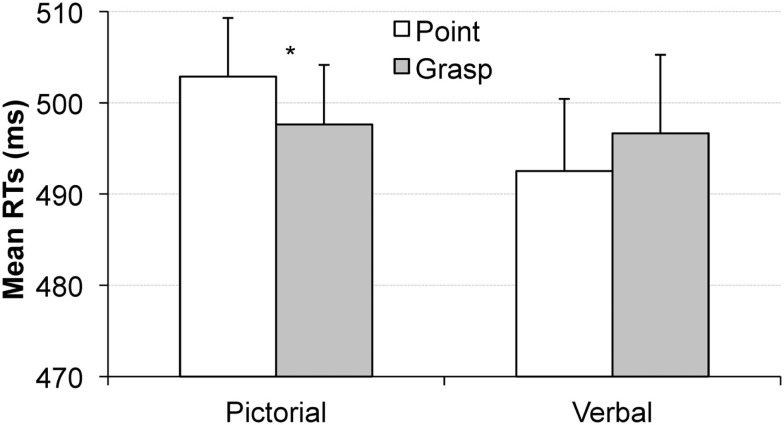
**Mean reaction times in the visual search task obtained in Experiment 1 as a function of pointing (white bars) or grasping (gray bars) for the pictorial cue condition (left) and the verbal cue condition (right)**. The congruency effect was observed for the pictorial cue condition but not the verbal cue condition. Error bars represent standard errors adjusted to within-subject designs, calculated according to the procedure described in Cousineau (2005).

The analysis on RT data showed a significant interaction of movement type and cue type, *F*(1, 16) = 8.9, *p* < 0.01, η*_p_*^2^ = 0.36, revealing that the effect of movement type depended on the type of cue (picture vs. word). This effect did not depend on the order of the tasks (interaction with the within-subject factor of task order: *p* > 0.4) or the order of the cue-blocks (interaction with the between-subject factor of cue type order: *p* > 0.8). Separate analyses revealed that in the pictorial cue condition, the movement type effect was significant, *F*(1, 17) = 4.4, *p* < 0.051, η*_p_*^2^ = 0.2, indicating faster RTs in the grasping condition than in the pointing condition (Δ*M* = 6 ms), see Figure 4, left. In the word cue condition, the movement type effect was not significant, *p* > 0.15 (Δ*M* = 4 ms with pointing condition eliciting slightly faster RTs than the grasping condition, see Figure 4, right).

The analysis of error rates in the search task showed neither a significant effect of movement type, *p* > 0.1 nor an interaction with cue type, *p* > 0.6. However, the pattern of error rates was in line with RT results: the grasping condition yielded less errors (*M* = 5.2%) than the pointing condition (*M* = 5.7%), which was more pronounced in the pictorial cue condition (Δ*M* = 0.6%), as compared to the word cue condition (Δ*M* = 0.2%). The effect of target type was significant, *F*(1, 16) = 18, *p* = 0.005, η*_p_*^2^ = 0.53, indicating more misses (*M* = 8%) than false alarms (*M* = 2.3%), which suggests that participants adopted a more conservative rather than liberal strategy in target detection.

The analysis of error rates in the movement task revealed that picture cues yielded a somewhat larger error rate (*M* = 2.8%) than word cues (*M* = 1.5%), *F*(1, 17) = 4.9, *p* < 0.05, η*_p_*^2^ = 0.22. However, this was only observed when the movement task was performed after the search task, *F*(1, 17) = 5.3, *p* < 0.05, η*_p_*^2^ = 0.24, but not if tasks were performed in the opposite order, *p* > 0.08.

### Discussion

The goal of Experiment 1 was to examine whether various types of action representations would differentially affect perceptual processing. Similarly to Wykowska et al. (2009), participants performed a visual search task together with a movement task. This time, however, the to-be-performed movement was signaled either by a picture cue (like in Wykowska et al., 2009, 2011), or by a word cue. We reasoned that if the type action representation influences the (likelihood of) intentional weighting of processing perceptual dimensions, then we should observe differential impact of the types of cues on the congruency effects. This would indicate that the mental representation of an action evoked by observing an image of the action is more directly linked with the intentional weighting mechanism, than the representation triggered by verbal cues. This is presumably due to the fact that an image of an action bears a more direct relationship to the perceptual dimensions the given action depends on.

Results of Experiment 1 showed that the congruency effect depended on the type of cue, i.e., it was observed only in the picture cue condition but not in the word cue condition, and it was independent of whether the visual search task was performed first or second. Interestingly, the difference between picture cues and word cues is mainly due to the incongruent (pointing) condition rather than the congruent (grasping) condition. This might suggest that in some cases, the congruency effects reflect a conflict in the incongruent trials between the dimension primed by the movement cue and the dimension presented in the visual search task, rather than facilitation in the congruent condition. This possibility should be investigated in future experiments. Another interesting finding is that the task order did not modulate the congruency effects, even though the congruency effects were slightly more pronounced for the trials in which the movement was executed after the visual search task (see Footnote 1). This indicates that the action representation evoked during action planning processes is strongest before the action is executed but still remains activated for some time after action execution. This is in line with previous findings (Stevanovski et al., 2002; Oriet et al., 2003), which revealed that *blindness to response-compatible stimuli* (an effect reported originally by Müsseler and Hommel, 1997) occurred not only during action preparation/execution, but also for stimuli presented after the response was executed.

Most importantly for the purposes of this study, the results reveal that congruency effects are modulated by the type of cue that signals the required action type. This suggests that the nature of representation evoked by movement cues is crucial for inducing intentional weighting of perceptual dimensions. Interestingly, the analysis on error rates in the movement task suggested that participants prepared actions equally efficiently in response to either movement cue when the task did no longer require the maintenance of the movement representation, i.e., when the search task was performed after the movement task. However, when the search task was performed before the movement task (standard condition), more movement errors were committed in the picture than in the word cue condition. This might indicate that a visual representation of the to-be performed movement is slightly shorter-lasting than a semantic/verbal representation – which would fit estimates of the temporal capacity of visual vs. auditory short-term memory stores (Coltheart, 1980). Still, it is the pictorial condition that yielded the congruency effect. Therefore – as word cues did not yield larger error rates in the movement task – the lack of congruency effects in the word cue condition cannot be due to that word cues were less efficient in activating an action plan/motor program. Hence, taken altogether, the results of Experiment 1 suggest that the type of action representation has an impact on triggering or informing the intentional weighting mechanism.

## Experiment 2

In Experiment 1 we tested whether the nature of action representation, evoked by various types of movement cues, would have an impact on the intentional weighting mechanism. Results showed that, indeed, intentional weighting is more likely induced by action representation if triggered by an image of that action, as compared to a verbal representation. The aim of Experiment 2 was to replicate this observation with the use of two perceptual dimensions (similarly to the original study of Wykowska et al., 2009) and under conditions that made the word cues more informative with respect to *invariant* characteristics of the to-be-prepared action. In Experiment 1, word cues might have carried less information concerning the required action in general. Hence, representation of the required action might have been less rich than in the case of the pictorial cues, not only in terms of variable parameters but also with respect to invariant characteristics. The aim of Experiment 2 was to circumvent this by making the two types of cues equally informative in terms of *invariant* characteristics, and by making the pictorial cues be richer than word cues only with respect to action-specific parameters (e.g., the size and location of the to-be grasped cup). That is, we enriched the word cues by specifying that participants should point to/grasp the center of a cup, thereby verbally specifying the same invariant information that was carried by picture cues.

### Method

#### Participants

Twenty five paid volunteers (12 women) aged from 19 to 30 years (mean age: 24.4) took part in Experiment 2. None of the participants took part in Experiment 1. All but two were right-handed; all of them reported normal or corrected-to-normal vision. The experiment was conducted with the understanding and consent of each participant.

#### Stimuli and apparatus

Stimuli were presented on a 17″ CRT screen (100 Hz refresh rate) placed at a distance of 110 cm from an observer. Stimulus presentation was controlled by E-Prime presentation software (Psychology Software Tools, Pittsburgh, PA, USA). The to-be prepared action (i.e., grasping or pointing) was specified by either a picture cue or a word cue. The picture cues consisted in photographs of a left hand performing a pointing or grasping movement on a white paper cup, see Figure 4. The photographs were black and white covering 12.9° × 10.2° of visual angle.

Importantly, the word cues specifying what type of action to prepare (i.e., grasping or pointing) were more informative in terms of invariant characteristics than in Experiment 1. That is, they consisted of a centrally presented message in German “GREIFE EINEN BECHER MITTIG” (grasp a cup in the middle) or “ZEIGE AUF EINEN BECHER MITTIG” (point at a cup in the middle) covering 9.6° × 0.4° of visual angle in the first case or 10.7° × 0.4° in the latter case (each letter was of 0.4° × 0.4° size).

Similarly to Experiment 1, the search display always contained 28 items (gray circles, 1.1° in diameter; 22 cd/m^2^ of luminance) positioned on three imaginary circles with a diameter of 3.4°, 7.4°, and 11.3°, see Figure 2. The target was defined by either size (Figure 2B): a larger circle, 1.4° in diameter, or lighter luminance (53 cd/m^2^), see Figure 2C, and could appear on one of six lateralized positions (three left, three right) on the middle circle. Participants were to detect the target in target present trials (50% of trials, one mouse key) and reject blank trials (50% of trials, the other mouse key).

The MED was substituted with only three cups positioned linearly below the computer screen (see Figure 4), 70 cm in front of the observers, to allow for easy reach. There were three different cups: a small white (3 cd/m^2^) cup, 5 cm (2.8°) in diameter in the middle point; a middle gray (1.8 cd/m^2^) cup, 6.5 cm (3.7°) in diameter in the middle point; and a large dark gray (0.43 cd/m^2^) cup, 8 cm (4.5°) in diameter in the middle point. They were all equal in height (4.5°) and weight (2 g). Instead of an LED lighting up behind one of the cups (Experiment 1), in Experiment 2, a yellow asterisk (0.5°, R: 255, G: 211, B: 32 in the RGB scale) presented on the computer screen for 300 ms signaled which cup should be grasped/pointed to. The asterisk could appear at one of three different positions on the screen (10.0° below an imaginary horizontal axis in the middle of the screen and 4.5°, 11.3°, or 17.7° from the left border of the screen).

#### Procedure

All participants took part in three sessions, one practice session and two subsequent experimental sessions with at least 1 day, but not more than 2 days in between each of the sessions. In the practice session, participants performed two blocks of one movement type only (pointing or grasping, 48 trials per each block) and one block of both types of movement randomly intermixed (30 trials). Each of the experimental sessions proper consisted of 240 trials for each of the target dimensions, which is equal to 480 trials per session. At the beginning of the experimental session, participants performed two short warm-up blocks, one in which they practiced the movements only (36 trials) and one in which they practiced the movement task with the search task (18 trials). The movement type (point vs. grasp) was randomized. The target dimension was blocked and participants were instructed before each block whether they should search for a luminance or a size target. Each experimental session consisted of one block with size targets and one block with luminance targets. The order of the blocks was counterbalanced across participants. The type of cue (words vs. pictures) was varied across experimental sessions. That is, in one session (on the second day after the movement practice session), participants performed the task with only word cues (or only picture cues) presented and then, in the subsequent session on a separate day, they performed the task with the other cues presented. The order of cue type was also counterbalanced across participants.

**Figure 5:**
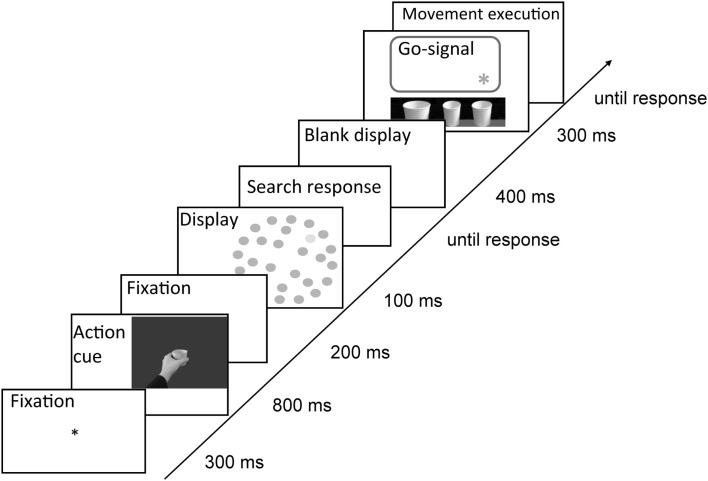
**Trial sequence in Experiment 2**. Either word or picture cues informed participants about the required action type. Participants always performed the search task first and only then the movement task. Target was defined either by size or luminance dimension. The movement was executed on one of the three linearly arranged cups beneath the computer screen. The exact cup which was supposed to-be grasped/pointed at was signaled by an asterisk.

To simplify the design, only one type of task order (visual search task first) was introduced in the trial sequence, as no interaction between task order and the effects of interest was obtained in Experiment 1. That is, in Experiment 2, the search task was to-be performed before movement execution, see Figure 5.

Therefore, in each trial, after a fixation display (300 ms), a cue (sentence or picture) was presented (800 ms), and then, subsequent to a blank display (200 ms), the search display was presented for 100 ms. Participants were asked to respond to the visual search task immediately by pressing the right/left mouse key to target present/absent displays with the index and middle finger of their right hand. Both speed and accuracy were stressed. After the response to the visual search task, and subsequent to a blank display (400 ms), the go-signal was presented for 300 ms, which signaled that observers should execute the prepared movement, i.e., to either point to or grasp the indicated cup with their left hand. Only accuracy was stressed in the movement task. Subsequent to the movement execution, a blank display was presented for 100 ms and a new trial started. Correctness of movement execution was registered by the experimenter monitoring the participants through a camera. For visualization of the trial sequence in Experiment 2, see Figure 5.

#### Data analysis

Incorrect movement trials as well as outliers in the search task (±3 SD from mean RT for each participant and each block) were excluded from further analyses. From the remaining data, RTs in the detection task were submitted to ANOVAs with: *cue type* (words vs. picture), *movement type* (point vs. grasp), *target dimension* (size vs. luminance), and *target presence* (target absent vs. target present trials) as within-subject factors. The order of *cue type* (word cues first vs. pictures first) was a between-subject factor. For the analyses of the error rates in the search task, incorrect trials in the movement task as well as outliers in the search task were excluded and individual mean error rates were submitted to analogous ANOVAs as in data analysis of RT data. Two participants were excluded from the analyses due to a high overall error rate (>45% in some conditions) and four participants were excluded due to longer overall reaction times relative to other participants (Overall Mean RT >550 ms; Overall Mean RT of the remaining participants: 390 ms, SD = 57 ms).

### Results

The analysis on mean RT data showed a significant interaction of movement type, target dimension, cue type, and target presence, *F*(1, 17) = 4.6, *p* < 0.05, η*_p_*^2^ = 0.21, revealing that the interaction between movement type and target dimension (as observed in Wykowska et al., 2009) depended on the type of cue (pictures vs. word cues) as well as target presence. This effect did not depend on whether the word cue condition or the picture cue condition was performed first (interaction with the between-subject factor of cue type order: *p* > 0.2).

Since the interaction between movement type and dimension was modulated by the type of cue, the subsequent analyses were conducted separately for picture cues and word cues.

#### Picture cue condition

In the picture cue condition, a 2 × 2 × 2 ANOVA with the factors movement type (point vs. grasp), dimension (size vs. luminance), target presence (present vs. absent), and a between-subjects factor cue type order (word cue first vs. picture cue first) revealed a significant interaction between movement type, dimension and target presence, *F*(1, 18) = 10, *p* < 0.01, η*_p_*^2^ = 0.36.

**Figure 6:**
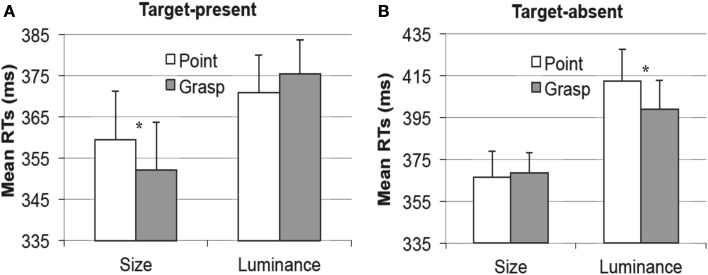
**Experiment 2, picture cue condition**. Mean reaction times in the visual search task, as a function of pointing (white bars) or grasping (gray bars) for the target present trials **(A)** and target absent trials **(B)**. Results for the size dimension are depicted on the left of **(A,B)** and for the luminance dimension [**(A,B)** right]. Error bars represent standard errors adjusted to within-subject designs, calculated according to the procedure described in Cousineau (2005).

In target-present trials, the interaction between movement type and dimension was significant, *F*(1, 18) = 5.2, *p* < 0.05, η*_p_*^2^ = 0.23, thereby replicating the congruency effects obtained by Wykowska et al. (2009), see Figure 6. Pair-wise comparisons between the pointing and the grasping conditions for size and luminance targets separately revealed that for the size targets, the congruent condition (grasping) elicited faster RTs (*M* = 352 ms) than the incongruent (pointing) condition (*M* = 360 ms), *t*(18) = 1.8, *p* < 0.05 (one-tailed), see Figure 6A, left. Luminance targets elicited a similar pattern showing slightly faster RTs in the congruent (pointing) condition (*M* = 371 ms) relative to the incongruent (grasping) condition (*M* = 376 ms), but this difference was statistically not significant, *p* > 0.2, see Figure 6A, right.

In target absent trials, the interaction between movement type and dimension was also significant, *F*(1, 18) = 5.5, *p* < 0.05, η*_p_*^2^ = 0.23 but the effects showed an opposite pattern: the congruent condition yielded longer RTs for the luminance dimension (*M* = 412 ms) relative to the incongruent condition (*M* = 399 ms), *t*(18) = 2, *p* < 0.05 (one-tailed, see Figure 6B, right), and an analogous (non-significant) pattern was observed for the size targets (ΔRT = 2 ms, *p* > 0.25), see Figure 6B, left.

#### Word cue condition

**Figure 7:**
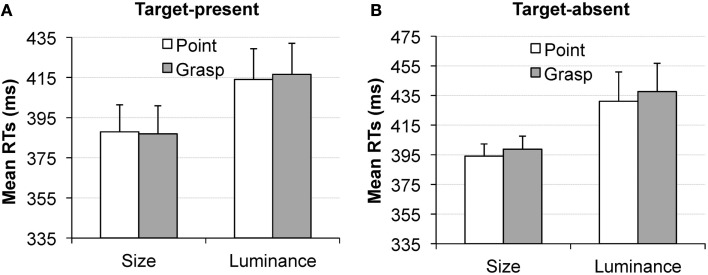
**Experiment 2, word cue condition**. Mean reaction times in the visual search task, as a function of pointing (white bars) or grasping (gray bars) for the target present trials **(A)** and target absent trials **(B)**. Results for the size dimension are depicted on the left of **(A,B)** and for the luminance dimension [**(A,B)** right]. Error bars represent standard errors adjusted to within-subject designs, calculated according to the procedure described in Cousineau (2005).

In the word cue condition, no main effects and no interactions approached the level of statistical significance, all *p*s > 0.24, see Figure 7. The differences between the pointing and grasping movement were equal to 1 ms in the size condition for target present trials (Figure 7A, left); 2.5 ms in the luminance condition for target present trials (Figure 7A, right); 4.7 ms in the size condition for target absent trials (Figure 7B, left) and 6 ms in the luminance condition for target absent trials (Figure 7B, right).

#### Error rates

Analogous analyses on error rates in the search task showed no significant effects, all *p*s > 0.07, *F*s < 3.7. However the interaction of cue type, dimension and movement type, was marginally significant, *F*(1, 17) = 3.6, *p* < 0.075. Subsequent analyses for each of the cue types separately did not reveal any significant effects or interactions of interest: in the picture cue condition, neither the interaction between dimension and movement type, *p* > 0.28 nor between target presence, dimension, and movement type was significant, *p* > 0.14; in the word cue condition, the interaction between dimension and movement type was also not significant, *p* > 0.14 and so was the interaction between target presence, dimension, and movement type, *p* > 0.57. The three-way interaction observed in the first analysis suggests only that the error rates were generally slightly lower for the grasping condition as compared to the pointing condition [(*M* = 4.3 vs. 4.5%) for size targets, picture cue condition; (*M* = 8.8 vs. 9.8%) for luminance targets, picture cue condition; and (*M* = 6.5 vs. 7%) for size targets, word cue condition] except for the luminance targets in the word cue condition, where the pointing movement elicited slightly lower error rates than the grasping movement (*M* = 10.5 vs. 11.4%). However, since none of the effects actually reached the level of significance, no conclusions can be drawn from the error rate data.

Analysis on the error rates in the movement task revealed that the type of cue (word vs. picture) did not influence movement performance, *t*(18) = 1.5, *p* > 0.15 with *M* = 2.7% for the picture cues and *M* = 3.8% for the word cues.

### Discussion

The aim of Experiment 2 was to examine whether the interaction between congruency effects and the type of cue (words vs. pictures) in Experiment 1 might have been due to the fact that the word cue carried less information than the pictorial cue about invariant characteristics of the to-be-prepared action. To do so, we enriched the word cues by specifying further also the invariant characteristics, i.e., by including the information that participants should grasp/point to a cup at its center. Thus, not only did the cues evoke the representation of the cup (together with the representation of the movement type) but also of a particular way in which the cup should be grasped/pointed to. This way, the word cues were assumed to-be equally informative as the pictorial cues with respect to invariant characteristics of the action, but not with respect to the varying parameters of the planned action. Furthermore, in Experiment 2, we introduced a second dimension of the visual search target (luminance), thereby making the results more directly comparable to the findings of Wykowska et al. (2009).

Despite these changes, Experiment 2 replicated the results of Experiment 1 by confirming that picture but not word cues induced an intentional weighting mechanism, which manifested itself through the action-perception congruency effects. That is, preparing an action that was congruent with a given perceptual dimension evoked better performance in a perceptual (visual search) task, as compared to when an incongruent action was being prepared. The congruency effects were particularly pronounced for target trials, and when the targets were defined by size. Interestingly, in target absent trials the congruency effects reversed, revealing faster RTs for the incongruent condition, as compared to the congruent condition. This pattern of results fits with a tendency already observed in Wykowska et al. (2009, Experiment 3) and might suggest that the intentional weighting of the action-relevant dimension impairs performance when a trial requires a negative response. This seems to be an intuitive consequence of such a weighting mechanism: if there is no target, one needs to suppress the (presumably) enhanced activation the pre-weighted dimension representation in order not to produce a false alarm. This suppression might produce an additional cost in performance. On the contrary, in target present trials, the pre-activation of the action-relevant dimension boosts the activity elicited by the presence of the target. Hence, detection of such a target is facilitated, which results in a performance benefit.

The finding that the congruency effects were more pronounced for the size than for the luminance dimension might be due to the fact that size targets were more salient than luminance targets. This is suggested by the main effect of dimension in the first analysis, *F*(1, 15) = 17.5, *p* < 0.005, η*_p_*^2^ = 0.53, which suggests that size targets were easier to detect (*M* = 377 ms) than luminance targets (*M* = 408 ms). This is plausible, since size targets were significantly larger in diameter than the distractors, and hence indeed popped-out from the context elements. Interestingly, in Wykowska et al. (2009, Experiment 3), congruency effects were more pronounced for the luminance targets than for the size targets and luminance targets were detected faster. This pattern suggests that the intentional weighting mechanism operates at the early, possibly pre-attentive stages of processing. The intentional weighting mechanism seems to enhance the saliency signal elicited by salient elements in a visual search array, which allows for efficient detection of salient targets. However, if targets are not salient enough and require more focused attention in order to-be detected, the intentional mechanism might be less potent.

In sum, and importantly for the purposes of this study, Experiment 2 replicated the findings of Experiment 1, confirming that action-perception congruency effects depend on the type of cue that triggers movement preparation. Supplemented by the finding that the word cues did not impair movement performance relative to picture cues – and hence were no less effective in activating motor programs – this suggests that the intentional weighting mechanism is more likely triggered by action representations that relate to action-specific parameters in addition to invariant characteristics of the action. This confirms the idea that the functional role of the intentional weighting mechanism is to provide information for open parameters of action control.

## General Discussion

Humans have developed an efficient way of optimizing their interaction with the surrounding environment by tuning perception to currently planned actions. An intentional weighting mechanism was postulated (e.g., Hommel et al., 2001a; Wykowska et al., 2009), which is assumed to increase the gain of output from perceptual dimensions (dimension maps) that are action-relevant – similarly to the functioning of other weighting or biasing mechanisms (e.g., Bundesen, 1990; Wolfe, 1994; Desimone and Duncan, 1995; Found and Müller, 1996; Reynolds et al., 1999; Müller et al., 2003; Wolfe et al., 2003). In line with ideomotor theorizing (e.g., Lotze, 1852; James, 1890; Greenwald, 1970; Prinz, 1987, 1997; Hommel et al., 2001a), the action-related weighting of perceptual information might allow the delegation of online action control to the environment. Action representation can thus be restricted to specifying a few goal-relevant action invariants and biasing attention toward feature dimensions that are likely to provide information that is able to specify the remaining action parameters online. For grasping actions, size and orientation are feature dimensions that are well suited to provide such information – by allowing the online control of grip aperture and hand orientation, while for pointing actions, location-specifying feature dimensions, such as luminance, are likely to-be more useful. Accordingly, one would expect that preparing and executing a grasping action biases attention toward size and orientation while preparing and executing a pointing action biases attention toward location and luminance. This is exactly what the findings of Fagioli et al. (2007) and Wykowska et al. (2009, 2011) demonstrate, as well as the present conditions with pictorial cues.

The present results extend previous findings by showing that word cues do not have the same impact on the congruency between actions and perceptual dimensions as picture cues. This is in line with the idea that picture cues trigger action plans that specify the invariant characteristics of the planned action and that additionally, they increase the output gain of action-relevant perceptual dimensions; while word cues do the former but not the latter. In other words, the intentional weighting mechanism, whose functional role is to provide information for open parameters during online action control (Figure 1, unfilled circle in “Action plan”), is more likely to-be activated by pictorial than by verbal representations of the to-be-planned action. This implies that congruency effects are produced by a mechanism of intentional weighting that operates on selection mechanisms in perceptual processing (Figure 1, see dashed line from the “Action Representation” module to the output of the dimension maps).

As witnessed by the absence of main effects of cue type, verbal action descriptions are sufficiently potent to support planning the right kind of action. We hypothesize that both pictorial and verbal cues support the planning of the intended, invariant characteristics of the planned action, such as the specification of the effector, the goal object, the action type, etc. In the present paradigm, such a representation should be sufficient to perform the action efficiently, since no (or not much) online adjustment is required. However, if online adjustment would be necessary, as in double-step tasks where the target location is modified after movement onset (e.g., Prablanc and Pélisson, 1990), it should be possible to demonstrate less efficient adaptation with word cues.

The present observation of differential impact of word and picture cues on congruency effects parallels findings reported in the literature on the visual attention. As mentioned earlier, several studies have shown that picture cues are more effective than word cues in establishing and maintaining target templates for visual search, which are considered to increase the top-down weighting of perceptual dimensions that support target discrimination (Wolfe et al., 2004; Vickery et al., 2005). By analogy, the pictorial cues of the present study can be considered to directly specify the necessary dimensions for online action control (e.g., size or location). This suggests that the same intentional weighting mechanism is responsible for biasing perceptual processing and fine-tuning action planning (Memelink and Hommel, 2012).

The framework we suggest fits with a number of previous suggestions. As already mentioned, Hommel et al. (2001a,b) have considered that both perceptual and action selection proceed by increasing the weights of task-relevant features. They also suggested that feedforward action planning proper is restricted to the invariant aspects of an action, while online sensorimotor loops are responsible for filling in open parameters. Along the same lines, Glover (2004) claimed that high-level perception and action planning proceed along offline ventral pathways while online action adjustments proceed via a dorsal action control pathway. A similar logic is applied by the Planning and Control Model (PCM) of motorvisual priming suggested by Thomaschke et al. (2012a,b) which, like Hommel et al. (2001a) and Glover (2004), distinguishes between action planning and movement control processes. Action planning integrates information concerning categorical representations of action features while movement control consists in representations of spatial feature dimensions of a given action and its goal. PCM predicts interference effects when perception and (planned) action share certain characteristics at the categorical level (due to code occupation by action planning), and facilitation effects when perception and action share spatial feature dimensions. Action planning processes, according to PCM, serve the purposes of specifying “action goals, situational factors, and knowledge about one’s own motor system into a consistent action plan” (Thomaschke et al., 2012b, p. 393) while the functional role of movement control is to fine-tune spatial characteristics of the action in order to “reduce any potential mismatch between the predicted course of the movement and momentary spatial target characteristics” (Thomaschke et al., 2012b, p. 393). Even though this model is currently restricted to the processing and selection of spatial information, the general logic underlying its architecture is consistent with the theoretical approach we suggest.

In sum, the present results support the idea that visual and verbal action cues trigger different types of action control (Tubau et al., 2007). This might be due to different modes of stimulus representation, as suggested by Proctor et al. (2009), which may use different modes of action planning. Visual cues depict the action that is to-be performed, and are thus likely to activate visual action representations or, more precisely, representation of how it looks to carry out the action. As we have discussed, ideomotor theory assumes that action planning is mediated by sensory representations of action-specific feedback (e.g., Greenwald, 1970; Prinz, 1997) and numerous findings have provided evidence that the activation of such sensory representations is often sufficient to activate the related action plans and motor structures (Jeannerod, 2003; Rizzolatti and Craighero, 2004; Schütz-Bosbach and Prinz, 2007) – more than symbolic cues can do (Iacoboni et al., 1999). Even though more research will be necessary to reveal the cognitive mechanisms underlying these different types, it seems clear that they do not bias attentional selection to the same degree.

## Conflict of Interest Statement

The authors declare that the research was conducted in the absence of any commercial or financial relationships that could be construed as a potential conflict of interest.

## Footnotes

^1^Note that two different task orders were introduced only to make sure that participants would prepare the required action immediately after the presented cue (and that the action representation would be activated while the visual search task was being performed). Hence, we did not have particular hypotheses concerning the task order factor. Yet, in order to test if there are no effects related to when the visual search is performed before or after the movement, we included this factor into the analyses. Even though the interaction between task order, cue type and movement type was not significant, separate analyses for each of the task order separately showed that the interaction of cue type and movement type was significant in the trials in which the search task was performed before the movement task (trial type I), *F*(1, 17) = 5.3, *p* < 0.05, η*_p_*^2^ = 2.4 and it did not reach the level of significance in trials in which the movement task was performed before the search task (trial type II), *F* < 2.5, *p* > 1.5. The pattern, however, was similar for both types of trials, with faster mean RTs in the grasping condition (trial type I: 498 ms, trial type II: 497 ms), as compared to pointing (trial type I: 504 ms, trial type II: 502 ms) in the pictorial cue condition, and a tendency for a reversed effect in the word cue condition (trial type I: 490 ms in pointing vs. 496 ms in grasping; trial type II: 495 ms in pointing vs. 497 ms in grasping).
